# Supplementary material for: Design, Synthesis, and Biological Evaluation of N-Acylhydrazones and Their Activity Against Leishmania amazonensis Promastigotes
Source: Microorganisms. 2025 Jul 2;13(7):1563. doi: 10.3390/microorganisms13071563 (PMC12298860; doi:10.3390/microorganisms13071563)
Supplement: Supplementary file 1 [file microorganisms-13-01563-s001.zip › HPLC amostras-20241114T144635Z-001.zip!/HPLC amostras/HPLC composto 15 - PMM 73 - 17.pdf]

Farmanguinhos - Fiocruz
Serviço de Métodos Analíticos

PMM 73 - 17.lcd
2018 - Sintese 1 - João Mafra - 2-5/2-28-4 - PMM 73 - 17.lcd
20/06/2018, 10:42:12

Sample Name	: PMM 73 - 17		
Sample ID	: 118716		
Sample Full Path	: 2018 - Sintese 1 - João Mafra - 2-5/2-28-4 - PMM 73 - 17.lcd		
Data Filename	: PMM 73 - 17.lcd		
Method Filename	: Nitroimidazol.lcm		
Batch File	: 2018 - Sintese 1 - João Mafra - CLAE-10 - CLAE 10_2018 _0619.lcb		
Vial #	: 1-15	Sample Type	: Unknown
Injection Volume	: 10 uL		
Date Acquired	: 20/06/2018 10:42:42	Acquired by	: Marcio Soares
Date Processed	: 27/06/2018 08:48:36	Processed by	: Marcio Soares
Data Comment	: Método: Nitroimidazol		

Preparo da Amostra: 1,62 mg foram adicionados 4 mL de metanol, agitada em vortex e levada ao ultrassom por 3 min. Posteriormente filtrada em membrana PVDF de 0,22µm e injetada.

Method

<<Header>>

<<Pump>>
Total Flow : 1,0000 mL/min
Pump A : LC-20ADXR

<<Mobile Phase Name>>
Pump A Mobile Phase A : Água
Pump A Mobile Phase C : Acetonitrila

<<LC Time Program>>

Time	Module	Command	Value	C
0,01	Pumps	Solvent C Conc.	20	
0,01	Pumps	Total Pump A Flow	1	
5,00	Pumps	Solvent C Conc.	20	
22,00	Pumps	Solvent C Conc.	90	
25,00	Pumps	Solvent C Conc.	90	
33,00	Pumps	Solvent C Conc.	20	
39,00	Pumps	Solvent C Conc.	20	
39,01	Controller	Stop		

<<Data Acquisition>>
LC Stop Time : 39,01 min

<<Oven>>
Oven Temperature : 30 C

Instrumentation

<<Instrument>>

<<Pump A>>
Pump A Type : LC-20ADXR

<<System Controller>>
SCL Type : CBM-20A

<<Autosampler>>
Autosampler : SIL-30AC

<<Oven>>
Oven Type : CTO-20AC

<<Column>>
Column Name : Supelcosil LC- 8 - 5 µm
Column ID : CL 0173
Length : 150 mm
Internal Diameter : 4,6 mm
Max Number of Injections : 9999
Expiration Date : 28/04/2015
Max Temperature : 80,0 C
Installation Date : 28/04/2030

<<PDA>>
Detector Name : PDA
Detector Type : SPD-M20A

<<Degasser>>
Degasser : DGU-20A5R

Farmanguinhos - Fiocruz
Serviço de Métodos Analíticos

PMM 73 - 17.lcd
2018 - Sintese 1 - João Mafra - 2-5/2-28-4 - PMM 73 - 17.lcd
20/06/2018, 10:42:12

Chromatogram

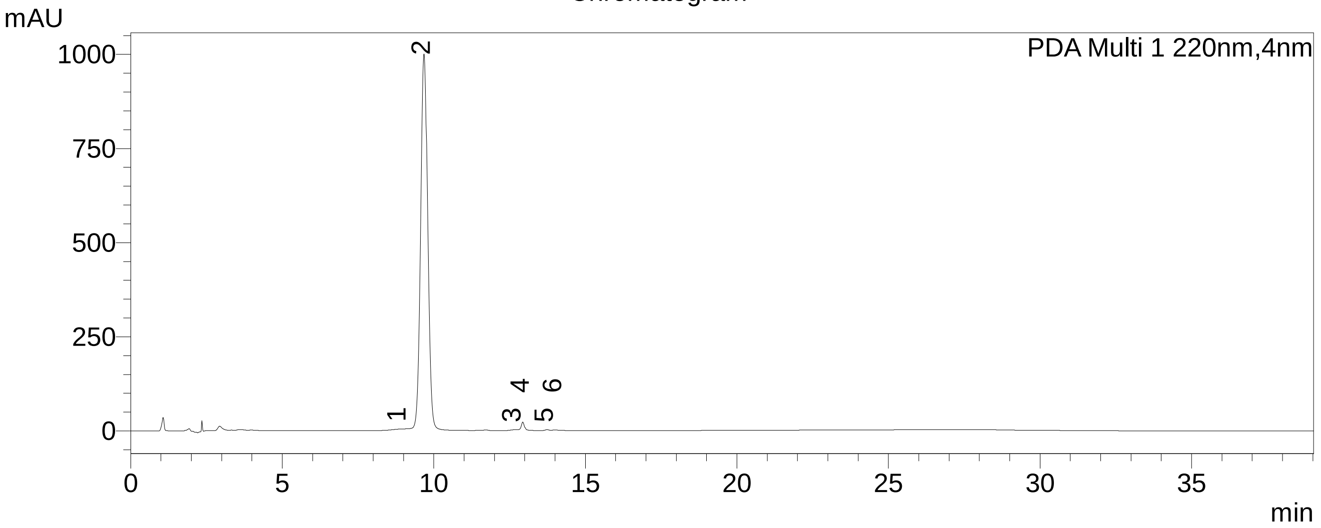

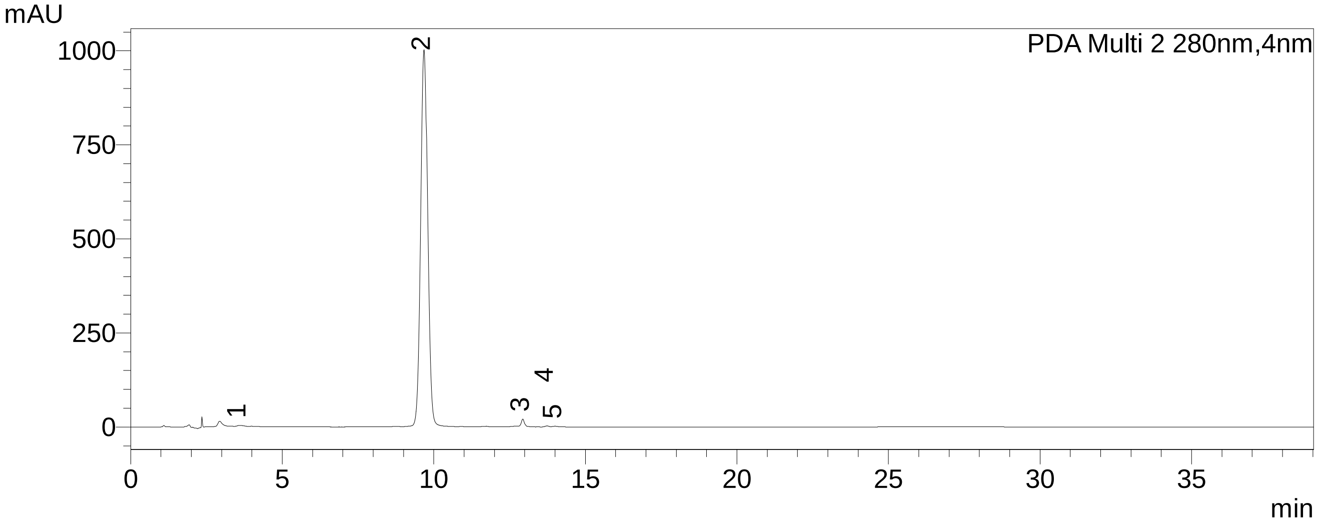

Peak Table

PDA Ch1 220nm

Peak#	Ret. Time	Name	Area	Area%	Theoretical Plates/meter(USP)	Tailing Factor	Resolution(USP)	Capacity Factor(k')
1	8,87		103443	0,6	--	--	--	--
2	9,68		16026510	98,0	54488	1,044	--	0,091
3	12,67		29757	0,2	34934	--	5,301	0,428
4	12,94		155522	1,0	429264	--	0,593	0,458
5	13,74		20346	0,1	348730	--	3,596	0,548
6	14,01		13646	0,1	287229	--	1,055	0,578
Total			16349225	100,0				

PDA Ch2 280nm

Peak#	Ret. Time	Name	Area	Area%	Theoretical Plates/meter(USP)	Tailing Factor	Resolution(USP)	Capacity Factor(k')
1	3,59		36067	0,2	8513	1,421	--	--
2	9,68		15984103	98,7	54838	1,050	14,691	1,696
3	12,94		135250	0,8	465457	1,078	10,466	2,603
4	13,74		21797	0,1	352057	--	3,671	2,825
5	14,01		13824	0,1	279605	--	1,051	2,900
Total			16191040	100,0				
